# Uncovering transport, deposition and impact of radionuclides released after the early spring 2020 wildfires in the Chernobyl Exclusion Zone

**DOI:** 10.1038/s41598-020-67620-3

**Published:** 2020-06-30

**Authors:** Nikolaos Evangeliou, Sabine Eckhardt

**Affiliations:** Department of Atmospheric and Climate Research (ATMOS), Norwegian Institute for Air Research (NILU), Instituttveien 18, PO Box 100, 2027 Kjeller, Norway

**Keywords:** Environmental sciences, Environmental impact

## Abstract

In the beginning of April 2020, large fires that started in the Chernobyl Exclusion Zone (CEZ) established after the Chernobyl accident in 1986 caused media and public concerns about the health impact from the resuspended radioactivity. In this paper, the emissions of previously deposited radionuclides from these fires are assessed and their dispersion and impact on the population is examined relying on the most recent data on radioactive contamination and emission factors combined with satellite observations. About 341 GBq of ^137^Cs, 51 GBq of ^90^Sr, 2 GBq of ^238^Pu, 33 MBq of ^239^Pu, 66 MBq of ^240^Pu and 504 MBq of ^241^Am were released in 1st–22nd April 2020 or about 1,000,000,000 times lower than the original accident in 1986 and mostly distributed in Central and East Europe. The large size of biomass burning particles carrying radionuclides prevents long-range transport as confirmed by concentrations reported in Europe. The highest cumulative effective doses (> 15 μSv) were calculated for firefighters and the population living in the CEZ, while doses were much lower in Kiev (2–5 μSv) and negligible in Belarus, Russia and Europe. All doses are radiologically insignificant and no health impact on the European population is expected from the April 2020 fires.

## Introduction

On April 4th, 2020, the Ukrainian authorities reported that a fire started at Volodymyrivka village located in the Chernobyl Exclusion Zone (CEZ). The fire damaged up to 20 ha of forest litter, before firefighters could stop it shortly after. The same day, it was reported that another fire had started in the Kotovsky forests burning 4 ha of forest litter^1^. On April 6th, 2020, actions to eliminate two more fires were reported again in Kotovsky Forest (25 ha), while on April 7th, 2020, measures were taken to extinguish a grass (6.5 ha) and a forest litter fire (4 ha) in the territory of the Kotovsky Forest in the CEZ. In the afternoon of the same day, a gust spread the grass fire across other regions of the Kotovsky forest. The fire spread to six regions of the Denysovets Forest in the CEZ^2^. On April 8th, grass and litter fires continued in the Kotovsky Forest, while another fire was observed in seven regions between Poliske and Volodymyrivka villages. The same day, another grass and litter fire near the Ososhnya village was eliminated (4 ha). On April 9th, firefighting activities were still ongoing near the Chistogolovka village with a smoldering fire burning over 20 ha. Several other smoldering fires that burned shrubs were reported in the Denysovetsky forest and in Poliske and Volodymyrivka vilages^3^. Firefighting activities continued the next five days in Korogodsky, Kotovsky and Denysovets regions; however, about 20 ha per day were burned. On April 14th, new fires were observed in the Parishovsky mountain, and in Janiv Lubianski approaching the city of Chernobyl. On April 15th, grass, cane and litter fires were reported in villages near the Poliske town, and in the territory of Korogodsky, Kotovsky, Denysovytsky, Paryshevsky and Lubyansky forests that continued burning the next days. On April 19th, attempts to extinguish fires in the territories of Korogodsky, Lubyansky, Paryshivsky, Dytyatkovsky and Dennytsky were ongoing, while on April 21st, new smoldering fires ignited in Denysovsky, Korotnysky and Kotovsky, and peat fires in Chapayevka and Polisko^4^. After April 22nd, clouds covered the CEZ making fire detection difficult^5^. In all cases, radiation background remained within normal limits in Kiev (Kiev < 0.013 mR h^−1^, Kiev region < 0.011 mR h^−1^), as well as in Chernobyl (0.021 mR h^−1^ at control permittable level of up to 0.055 mR h^−1^). The fires continued to burn in May 2020 more debilitated.

Media coverage^6–8^ and speculations on the resuspended amounts of the radionuclides^9–11^ that are deposited in the CEZ since the Chernobyl Nuclear Power Plant (CNPP) accident in 1986 caused public concerns about the risk of artificial radioactivity on the local and European population. It has been previously suggested that resuspension after fires is a real fact in the CEZ^12–14^ and strongly depends on the level of contamination rather than the amount of biomass to be burned^15^. It will become a more important problem in the future^16^, due to the pronounced increase of surface temperature that will, in turn, lead to more frequent drought events and to more fires in the area^17^.

Here, for the first time, the emissions of the resuspended radionuclides (^137^Cs, ^90^Sr, ^238^Pu, ^239^Pu, ^240^Pu and ^241^Am) after the April 2020 fires in the CEZ are quantified using satellite measurement, and their respective transport is modelled. Furthermore, a health assessment is performed on four population groups with respect to the total effective doses committed after exposure to smoke plumes. These calculations are based on the most recent updates on the radioactive contamination of the CEZ and emission factors for radionuclides released from biomass burning combined with satellite data. All model results are compared to measurements reported all around Europe^18–21^ proving that the April 2020 fires in the CEZ were not only of local and regional interest.

## Results

All the results presented below are the average of three simulations using emissions of six radionuclides calculated with each of the three different methods (see Supporting Information). For each of the radionuclides in each simulation, three size distributions (< 2.5 μm, 2.5–10 μm, > 10 μm) have been considered as explained in “Methods” section.

### Emission, transport and deposition of radionuclides

**Figure 1:**
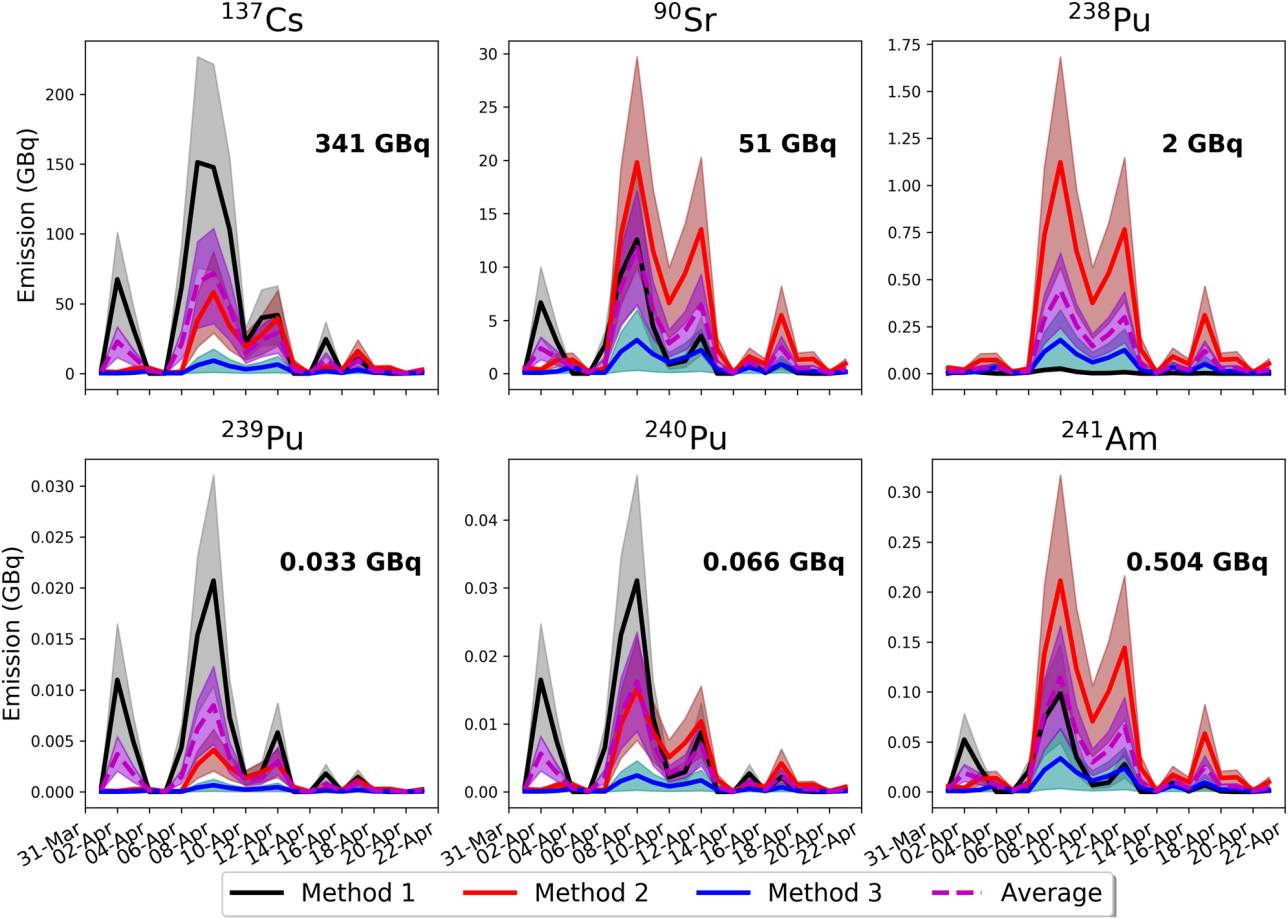
Daily emissions of radionuclides released from biomass burning in the CEZ during April 2020 (1–22 April) constrained with three different methods based on satellite data (Supporting Information). The daily average emissions from three methods are also given with the dashed line. Average total emissions of ^137^Cs, ^90^Sr, ^238^Pu, ^239^Pu, ^240^Pu and ^241^Am were calculated to be 341 GBq, 51 GBq, 2 GBq, 33 MBq, 66 MBq and 504 MBq, respectively. Uncertainties have been calculated as the standard deviation of the emissions using five different EFs for ^137^Cs (Table S3) and were extrapolated to the other radionuclides. Maps have been generated with the open access module matplotlib^63^ (license: https://matplotlib.org/3.2.1/users/license.html).

The daily emissions of the six radionuclides constrained with the three different methods (see Supporting Information) are shown in Fig. 1. In total, 341 GBq of ^137^Cs, 51 GBq of ^90^Sr, 2 GBq of ^238^Pu, 33 MBq of ^239^Pu, 66 MBq of ^240^Pu and 504 MBq of ^241^Am were released between 1st and 22nd April 2020. The French Institute of Radioprotection and Nuclear Safety (IRSN) has mentioned that the releases of ^137^Cs after the April 2020 fires in the CEZ should be around 200 GBq and has recently upgraded them to 700 GBq^21^, though without giving any explanation on the methodology used in these estimates^18^. These amounts are more than 1 billion times lower than the original accident of Chernobyl in 1986 (1 EBq = 10^19^ Bq)^22^. For comparison fire releases in the area, emissions of about 10,900 GBq of ^137^Cs, 1,500 GBq of ^90^Sr, 7.8 GBq of ^238^Pu, 6.3 GBq of ^239^Pu, 9.4 GBq of ^240^Pu and 29.7 GBq of ^241^Am had been estimated for the two fire events in the CEZ 5 years ago^14^. The large difference between the 2015 and 2020 events is not due to the larger severity of the 2015 fires, but rather to more accurate knowledge on the resuspension of radionuclides and their size distribution that we have nowadays, due to more targeted research (Method 1, Supporting Information).

Surface activity concentrations (at altitudes < 100 m) of all six radionuclides every three hours are shown in Video 1. The plume was initially transported eastwards until April 5th, where it shifted south and then to the west having in most cases undetectable concentrations. The highest calculated emissions that took place on April 9th (Fig. 1) were pushed to the south, towards the Black Sea (April 10th) and later on to the Balkan countries and Northern Greece (April 11th–13th) in detectable concentrations (for ^137^Cs). Since then, the radioactive plume weakened substantially and was in concentrations of the order of μBq m^−3^ only in the close vicinity of the CNPP. A few concentration pulses appearing at the surface of West Russia (April 4th at 09:00) and in Turkey (April 10th at 09:00) are attributed to cold and more dense air coming from North Europe mixing with radionuclides from higher altitudes drifting them to the surface. In most cases, ^137^Cs was the only radionuclide that could be quantified due to its low detection limit in air samples (of the order of μBq m^−3^)^23,24^. Although ^90^Sr concentrations were in the same levels as those of ^137^Cs, its detection limit in air samples remains in the order of mBq, due to its complicated separation and measurement^25^. The rest of the radionuclides were in much lower concentrations (Video **1**).

**Figure 2:**
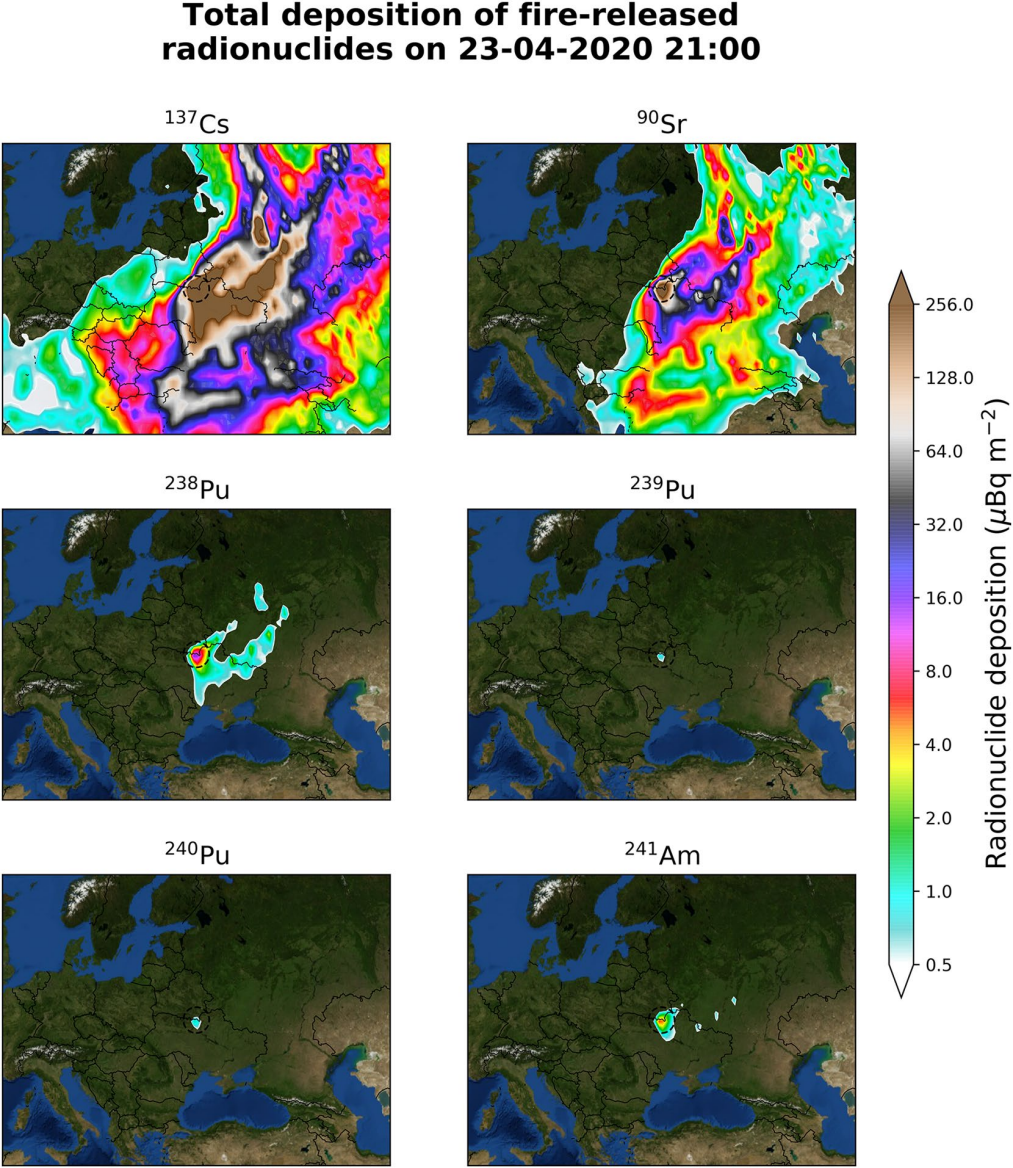
Total cumulative deposition of ^137^Cs, ^90^Sr, ^238^Pu, ^239^Pu, ^240^Pu and ^241^Am calculated with FLEXPART model after resuspension and transport, as a result of the April 2020 fires in the CEZ. It is evident that the resulting dispersion is rather insignificant for the European population with most of the deposition to be below the respective detection limits. Maps have been generated with the open access module matplotlib^63^ (license: https://matplotlib.org/3.2.1/users/license.html).

The evolution of the cumulative deposition of ^137^Cs, ^90^Sr, ^238^Pu, ^239^Pu, ^240^Pu and ^241^Am can be seen in Video 2 and in a detailed map in Fig. 2. The calculated deposition clearly indicates a regional event with the highest deposition of radioactive particles in the close vicinity of the plant. Most of the values presented in Fig. 2 are practically unmeasurable as the limit of detection for ground measurements is a few mBq kg^−1^ depending on sampling and measuring time^26,27^. Most soils have dry density between 1.1 and 1.6 g cm^−3^ (1.1–1.6 × 10^3^ kg m^−3^). Assuming that the sample was taken from an average depth of 10 cm (0.01 m), the limit of detection per area units becomes equal to a few hundreds of mBq m^−2^.

### Model response (validation) to reported measurements of ^137^Cs

**Figure 3:**
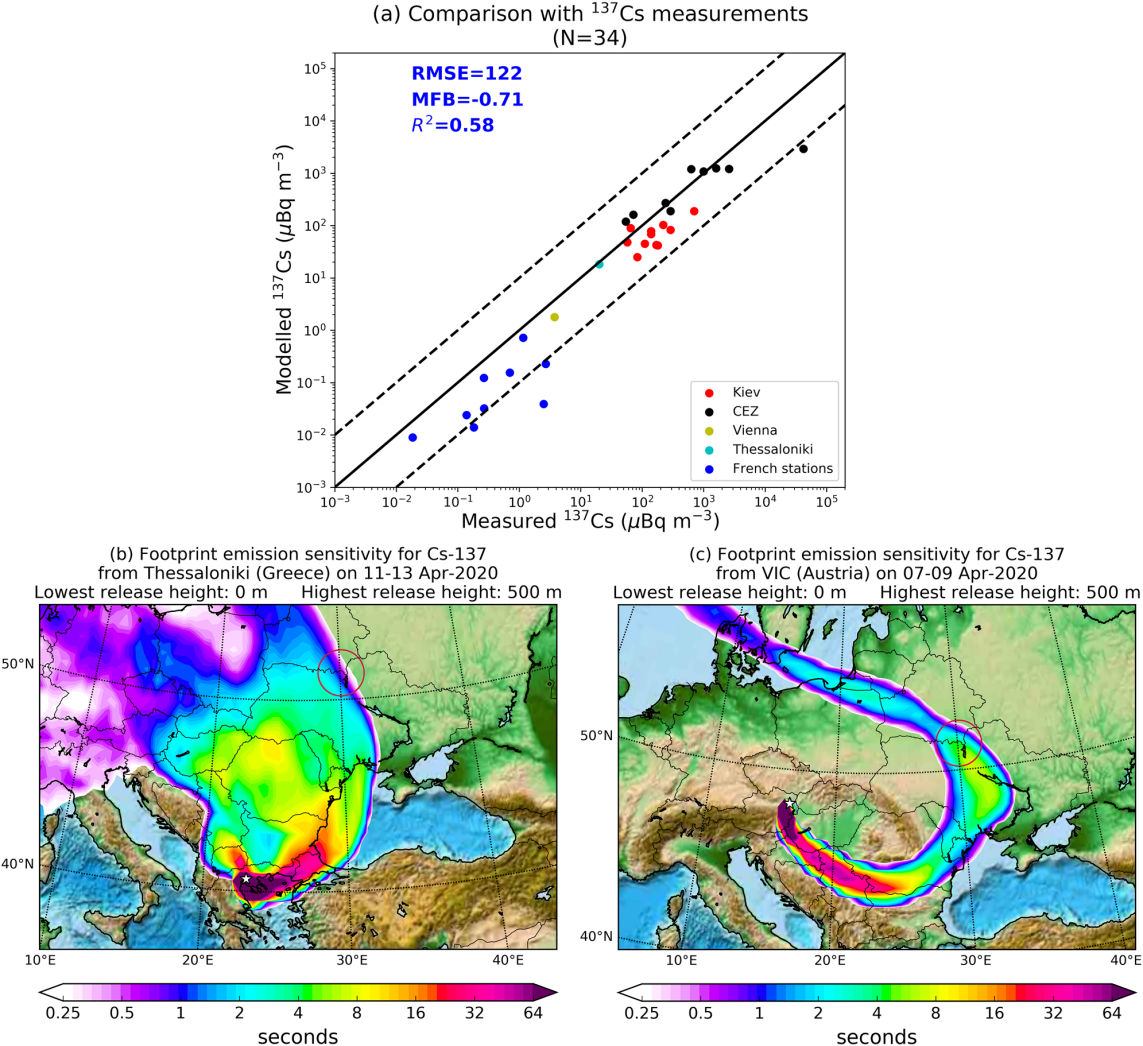
(**a**) Scatter plot of modelled against measured concentrations of ^137^Cs over Europe reported after the April 2020 fires in the CEZ. The 1 × 1 line and the upper and lower tenfold limits are also shown (full and dashed lines). Each set of measurements is coloured differently, namely, Kiev, CEZ, Vienna, Thessaloniki and French stations (Table S1). (**b**, **c**) Example footprint emission sensitivities for ^137^Cs from the two receptor points of Vienna and Thessaloniki, which reported measurable concentrations. Both clearly show that the air at the time of the measurement originated from the CEZ (red cycle). Maps have been generated with the open access module matplotlib^63^ (license: https://matplotlib.org/3.2.1/users/license.html).

In order to evaluate whether or not the radioactive plume released from the April 2020 fires in the CEZ is insignificant, with respect to its impact to living organisms, and our modelled emissions and transport accurate, a validation of modelled surface activity concentrations against observations of ^137^Cs reported by European groups and were published by the IRSN^18,21^ (Table S1) was attempted. The comparison (Fig. 3a) shows a Pearson coefficient of 0.58 that implies that the arrival times of the plume to the measurement stations were captured by the model. The model underestimates measurements with a mean fractional bias (MFB) equal to − 71%, while the root mean square error (RMSE) was estimated to be 122 μBq m^−3^ in a range of values between 4 and 180,000 μBq m^−3^ (see Supporting Information for the definitions of the statistical tests used). An MFB of − 71% means that the average modelled concentration is almost half of the average measured one (see Supporting Information). This underestimation may have a threefold explanation; (1) the emissions used for the present assessment may be biased low, (2) the mass fraction associated with each particle size in the model (< 2.5 μm, 2.5–10 μm, > 10 μm) may be wrong and (3) the model does not account for resuspension of previously deposited ^137^Cs by strong winds or by the construction-works made by the Ukrainian authorities to create fire breaks, in order to prevent further spread of the fires^1,2^. The latter has been already reported as an important factor of the increased background radiation in the CEZ^28,29^. It is noteworthy that stations close to the CEZ are captured by the model effectively and in some cases are overestimated, whereas stations far from the source are underestimated. This indicates that the assumption that exactly 60% of the particles was released in sizes > 10 μm following previous research^12,15,30^ is probably inaccurate. The size distribution has been found to depend on the type of the biomass burned and the type of fire (smoldering, flame or mixed)^31^. At this point, more precise evaluation for modelling purposes cannot be made, unless specific measurements have taken place. On the other hand, the fact that concentration levels were captured well proves that the use of such low EFs for radionuclides (1.2% for ^137^Cs, 0.2% for ^90^Sr and 0.1% for ^238–240^Pu and ^241^Am) resuspended after wildfires is largely realistic. For the measurements reported in Vienna International Centre and Thessaloniki that (Table S1) and are subject to long-range transport, the footprint emission sensitivities calculated with the retroplume mode of FLEXPART indicate that the airmasses at the time of the measurements originated from the CEZ (Fig. 3b,c).

## Discussion

### Model uncertainty

**Figure 4:**
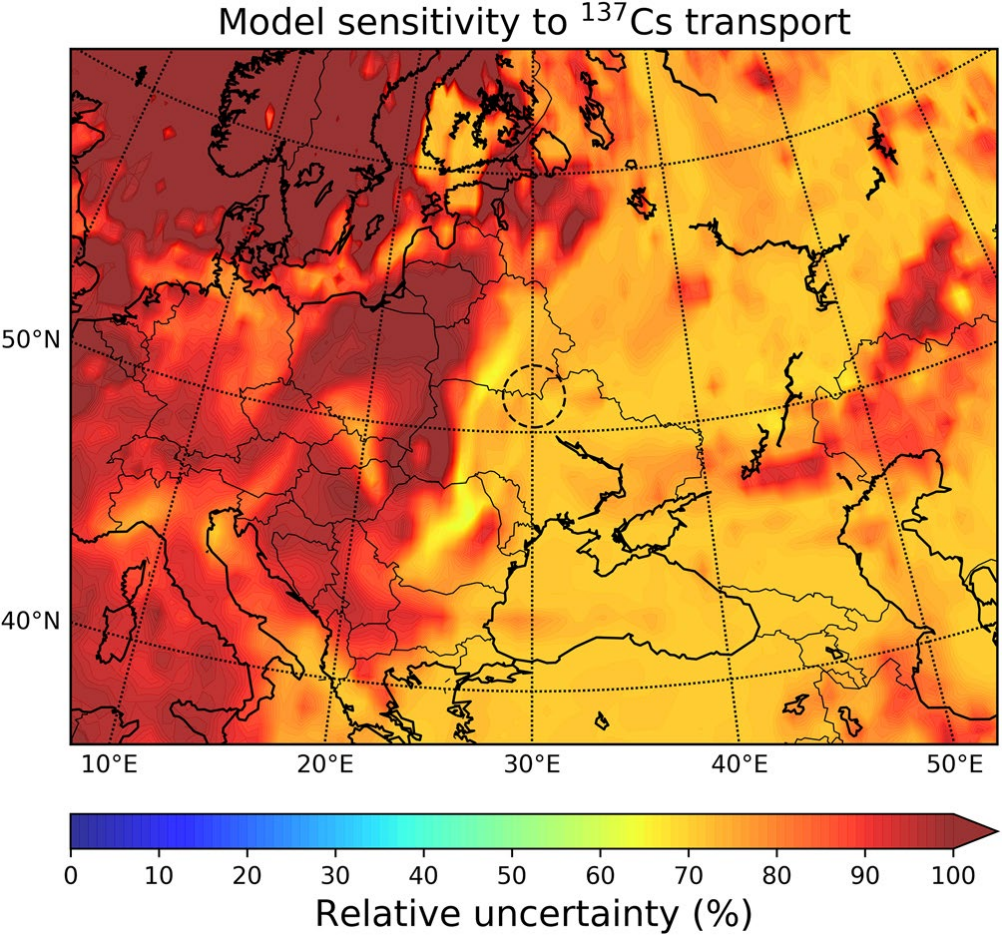
Relative model uncertainty of ^137^Cs transport and deposition after the April 2020 wildfires in the CEZ. The uncertainty was calculated as the standard deviation of total deposition from a 150-member model ensemble. The ensemble included three simulations with different emissions (709, 249 and 46 GBq), 10 combinations of mass fraction per particle size for each of them (Table S2) and five combinations of EFs (Table S3). Maps have been generated with the open access module matplotlib^63^ (license: https://matplotlib.org/3.2.1/users/license.html).

To estimate the model sensitivity of the aforementioned factors that have caused biased concentrations and considering that the model does not account for wind resuspension, we calculated the uncertainty of transport and deposition of ^137^Cs after the April 2020 fires in the CEZ. For this, a model ensemble that consists of three simulations with different emissions (709, 249 and 46 GBq) was used, 10 combinations of mass fraction per particle size, for each of them, always following the recommendation of Hao et al.^30^ that the majority of the emitted particles is at sizes > 10 μm (Table S2), and five different EFs (Table S3); this gives a total number of 150 (3 × 10 × 5) ensemble members. The model uncertainty (Fig. 4) was calculated as the standard deviation of ^137^Cs deposition resulting from all ensemble members. Model uncertainty appears to be about 70% near the source and increases with distance due to the focus on large particles^30^. Large particles deposit nearby due to gravitation and less likely reach center or west of Europe. This shows the large effect that the selection of size distribution has on dispersion modelling and denotes the importance to be defined prior to accurate simulation of transport after biomass burning events. Close to the source, the observed uncertainties are caused as a result of the perturbation of the EFs that result in large emission differences in the members of the ensemble.

### Cumulative effective doses

**Figure 5:**
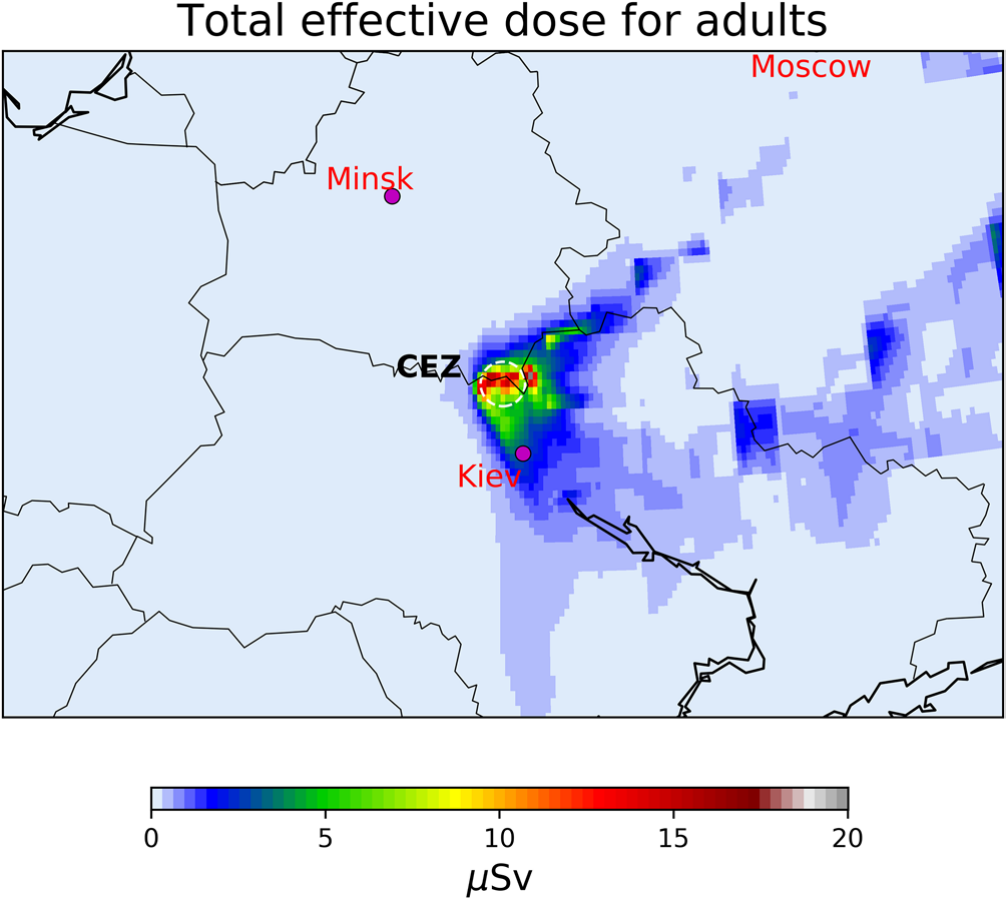
Total effective doses over Europe for the study period (1–22 April 2020) committed to adults. The exposure pathways of inhalation (internal), air submersion/immersion (external) and deposition (external) were examined, while food ingestion was omitted due to lack of information. The location of the capital cities of Ukraine (Kiev), Belarus (Minsk) and Russia (Moscow) are also shown together with the CEZ (30 km radium circle). Maps have been generated with the open access module matplotlib^63^ (license: https://matplotlib.org/3.2.1/users/license.html).

One of the most important aspects in the discussion of radioactive releases and transport is the health impact on the population. This attracts the greatest public attention and is often misused by the media. Here, we present the total effective doses committed to four population groups (1-year old infants, 10-year old children, adults and workers/firefighters) for the period 1st–22nd April 2020 (Figure S1). Effective doses appear to be very similar for all groups with slightly higher doses committed to infants due to the higher dose conversion coefficient used for all exposure pathways. The highest cumulative doses for April 2020 were calculated for the evacuated zone, where very few people nowadays live; therefore, we present only effective doses to adults. For adults and firefighters that stayed in the CEZ throughout the fire period (24 h of exposure was assumed with an occupancy factor indoors of 0.6, see Supporting Information), total effective doses were calculated to be 18 ± 8 μSv (Figure S1 and Fig. 5). Even with this extreme assumption that firefighters stayed in the CEZ, after trying to extinguish the fires, and then returned to extinguish another fire the next day staying 60% of the time indoors, the total doses received for April 2020 are about 1% of the annual external doses from background radiation due to the remained radioactive contaminants from the Chernobyl accident^32^. The highest contribution to the total effective dose was due to inhalation of radioactive fire smoke (72%). For an adult in Kiev the total effective dose from 22 days of exposure to the radioactive plume was 2–5 μSv. Doses for inhabitants of other major cities of the area such as Minsk (Belarus) or Moscow (Russia) are in the range of nSv and negligible in the rest of Europe. All doses are far below the annual threshold effective dose limit of 1 mSv established for members of the public in planned exposure situations (limits do not apply to existing or emergency exposure situations)^33^. Any health impact on the general local, regional or European population is not expected. The fires in the CEZ continued to burn after April 22nd, 2020 in significantly smaller intensities as seen in NASA’s EOSDIS^5^.

## Conclusions

In this study, the dispersion of radioactivity resulted after the April 2020 fires in the CEZ is quantified for the first time, in an attempt to settle down public concerns on the health effects of the dispersed radionuclides on the European population. Almost 400 GBq of ^137^Cs, ^90^Sr, ^238–240^Pu and ^241^Am were released between 1st–22nd April 2020. This is about 1 billion times lower than the emissions from the Chernobyl accident in 1986. The availability of measurements all over Europe assisted in the correct quantification of the emission levels and it is a step forward for future cases. The latter proved that radioactive particles produced from biomass burning are largely in the coarse mode and cannot reach far. A dosimetric assessment to different population groups and exposure pathways showed that the highest doses were committed to firefighters and people living in the CEZ (> 15 μSv) and were directly exposed to radioactive plumes, whereas doses decrease substantially with distance. All doses were far below the annual threshold limits for planned exposures to artificial radioactivity. No additional doses can be calculated for inhabitants of Belarus, Russia or the rest of Europe.

## Methods

In the recent times, the development of satellites has advanced our capacity to quantify biomass burning emissions. Several products are available, such as the Global Fire Emissions Database (GFED)^34^, the Fire Locating and Modelling of Burning Emissions (FLAMBE)^35^, the Fire INventory from NCAR (FINN)^36^, the Wildland Fire Emissions Information System (WFEIS)^37^ etc. However, they do not include radionuclide emissions mainly for two reasons: (1) natural occurring radioactive materials (NORM) are more significant in soil^38^ and radiologically insignificant in vegetation even in NORM hot-spot regions^39,40^ and (2) artificial radionuclides are only important in Chernobyl and Fukushima^41^. Hence, both NORM and artificial radionuclides are rather of regional interest.

Biomass burning emissions can be calculated in two different ways, (a) with the top-down approach using the FRP (Fire Radiative Power) seen from different satellites^42^ and (b) with the bottom-up approach based on the burned area, fuel loading, combustion completeness and biomass consumption^43^. Both methodologies require complicated retrieval algorithms, large data analysis and a lot of pre-processing that is time consuming; thus, prohibitive for rapid analysis and forecasting. Furthermore, they have been found to differ by a factor of 10^44^, due to burned area underestimation in satellites^45^ and static fuel loadings differences in fuel datasets^46^.

### Radionuclide emissions

In the present study, we have calculated emissions with three methods that are presented in detail in Supporting Information. Method 1 (bottom-up) uses burned area, based on a modified method from Stohl et al.^47^, and MODIS active fires (Figure S2) with the maximum confidence level (100%) combined with a statistical approach^48^. Then, we use the most recently updated emission factors (EFs) for radionuclides released from wildfires^30^ together with ground contamination data^49–51^ from the CEZ (Figure S3, Figure S4). Method 2 (top-down) combines EFs of ^137^Cs (in gr species per kg of dry mass burned) from Hao et al.^30^ with combusted biomass (in kg of dry mass) from Copernicus Atmosphere Monitoring Service (CAMS) Global Fire Assimilation System (GFAS)^52^ (Figure S4a). Finally, Method 3 (top-down) uses experimental ratios of ^137^Cs with particulate organic matter (POM) from Strode et al.^53^ for boreal regions and calculates emissions using POC from CAMS GFAS^52^ (Figure S5b).

### Altitude of the emissions

Injection height daily data were directly adopted from CAMS GFAS^52^. The data are simulated results of the Plume Rise Model (PRM)^54–56^ and have been previously used in simulations of radioactive releases from wildfires in Chernobyl^14,17^. To accurately model the dispersion of the radioactive smoke, we used the “altitude of plume bottom” and “altitude of plume top” in each release point.

### Atmospheric transport modelling

We simulated transport and deposition of ^137^Cs, ^90^Sr, ^238^Pu, ^239^Pu, ^240^Pu and ^241^Am with the Lagrangian particle transport model FLEXPART (Flexible Particle Dispersion Model) version 10.4^57^ and the calculated emissions from the three different methods. The model ran in forward mode from 1st–22nd April 2020 driven by 3-hourly 1 × 1 operational winds from the European Centre for Medium Range Weather Forecast. The spatial resolution of the output concentration and deposition fields was set to 0.5 × 0.5 in a global domain and the temporal every three hours. The model includes parameterisations of boundary layer turbulent mixing and convection processes that affect particle transport in clouds^57,58^, dry and wet deposition of aerosols^59^.

To identify the origin of the radioactive aerosol tracers arriving in specific regions (receptors), computational particles were released from the receptors and tracked 30 days backward in time in the so-called “retroplume” mode of FLEXPART. Thirty days should be sufficient time to include most aerosol emissions arriving at the receptor considering that a typical aerosol lifetime is about 1 week^60^.

It has been previously shown that labile ^137^Cs and ^90^Sr radionuclides are attached to sub-micron aerosol particles (< 1 μm)^61^, whereas refractory ^238^Pu, ^239^Pu, ^240^Pu and ^241^Am are PM10 particles^62^. Recently, Hao et al.^30^ reported that ^137^Cs released from laboratory fires was concentrated in the particulate fraction greater than 10 μm. To capture the reported size variability, we simulated all six species with a size distribution (characterised by a species diameter normalized logarithmic standard deviation^57^) around particle sizes of 0.25, 8 and 16 μm. Then, 60% of the airborne mass was associated to size distribution with a particle mean aerodynamic diameter of > 10 μm, 20% was associated to sizes between 2.5–10 μm and 20% to < 2.5 μm following Hao et al.^30^.

### Calculation of internal and external effective doses

A preliminary assessment on the committed internal (inhalation) and external (air submersion/immersion and deposition) exposure to radiation (^137^Cs) has been conducted for four population groups (1-year old infants, 10-year old children, adults and workers/firefighters). The methodology is based on the WHO^33^ report for Fukushima and includes the most recent updates on dose calculations. (see details in Supporting Information).

## Supplementary information

Supplementary information

Supplementary Video 1

Supplementary Video 2

## Data Availability

All satellite inputs used for the calculation of radionuclide emissions, FLEXPART simulation outputs and python scripts for all figures and videos are publicly available (10.5061/dryad.3bk3j9kgb) and can be directly accessed on DRYAD (https://datadryad.org/stash/share/LimJYhQWsbijkbU5yYl-ihcHC20aKiCdYSSGWIH4lyY) or upon request to the corresponding author.
