# Supplementary material for: Uncovering transport, deposition and impact of radionuclides released after the early spring 2020 wildfires in the Chernobyl Exclusion Zone
Source: Sci Rep. 2020 Jun 30;10:10655. doi: 10.1038/s41598-020-67620-3 (PMC7327000; doi:10.1038/s41598-020-67620-3)
Supplement: Supplementary file 1 — Supplementary information [file 41598_2020_67620_MOESM1_ESM.docx]

**Supporting Information**

**Uncovering transport, deposition and impact of radionuclides released after the early spring 2020 wildfires in the Chernobyl Exclusion Zone**

**Nikolaos Evangeliou*, Sabine Eckhardt**

NILU - Norwegian Institute for Air Research, Department of Atmospheric and Climate Research (ATMOS), Kjeller, Norway.

***** Corresponding author: N. Evangeliou ([Nikolaos.Evangeliou@nilu.no](mailto:Nikolaos.Evangeliou@nilu.no))

Fig. S 1: Total effective doses over Europe for the study period (1–22 April 2020) committed to four population groups (1-year old infants, 10-year old children, adults and workers/firefighters). The exposure pathways of inhalation (internal), air submersion/immersion (external) and deposition (external) were examined, while food ingestion was omitted due to lack of information. The location of the capital cities of Ukraine (Kiev), Belarus (Minsk) and Russia (Moscow) is also shown. High doses (>15 μSv) were only calculated for the vicinity of the CEZ. However, these doses are far below the annual threshold effective dose limit of 1 mSv established for members of the public in planned exposure situations (limits do not apply to existing or emergency exposure situations) ^1^. Maps have been generated with the open access module matplotlib^23^ (license: <https://matplotlib.org/3.2.1/users/license.html>).

**Methods**

**Method 1: Emissions derived from MODIS active fire – based burned area combined with ground contamination measurements and radionuclide EFs.** Near real-time (NRT) Moderate Resolution Imaging Spectroradiometer (MODIS) Thermal Anomalies/Fire locations - Collection 6 processed by NASA's Land, Atmosphere Near real-time Capability for EOS (LANCE) Fire Information for Resource Management System (FIRMS) are based on MOD14/MYD14 products. The thermal anomalies/active fires represent the centre of a 1km pixel that is flagged by the MODIS MOD14/MYD14 Fire and Thermal Anomalies algorithm ^2^ as containing one or more fires within the pixel. The product detects fires of 1000 m^2^ or less in cloud-free conditions. Detections depend on the time of the satellite overpass and other parameters and the overall efficiency is highlighted by confidence levels (0–100%) for every fire detection. Here, we took into account only these detections with the maximum confidence level (100%), in contrast to Stohl et al.^3^ who used fire detections with confidence level above 75% (**Fig. S 2**). Following the reports of the State Emergency Service of Ukraine (<https://www.dsns.gov.ua/en/Dovidka-za-dobu/>), we assumed a burned area of 30 ha per pixel, which is a very conservative assumption considering a value of 180 ha per fire pixel reported from Wotawa et al.^4^ for boreal forest fires.


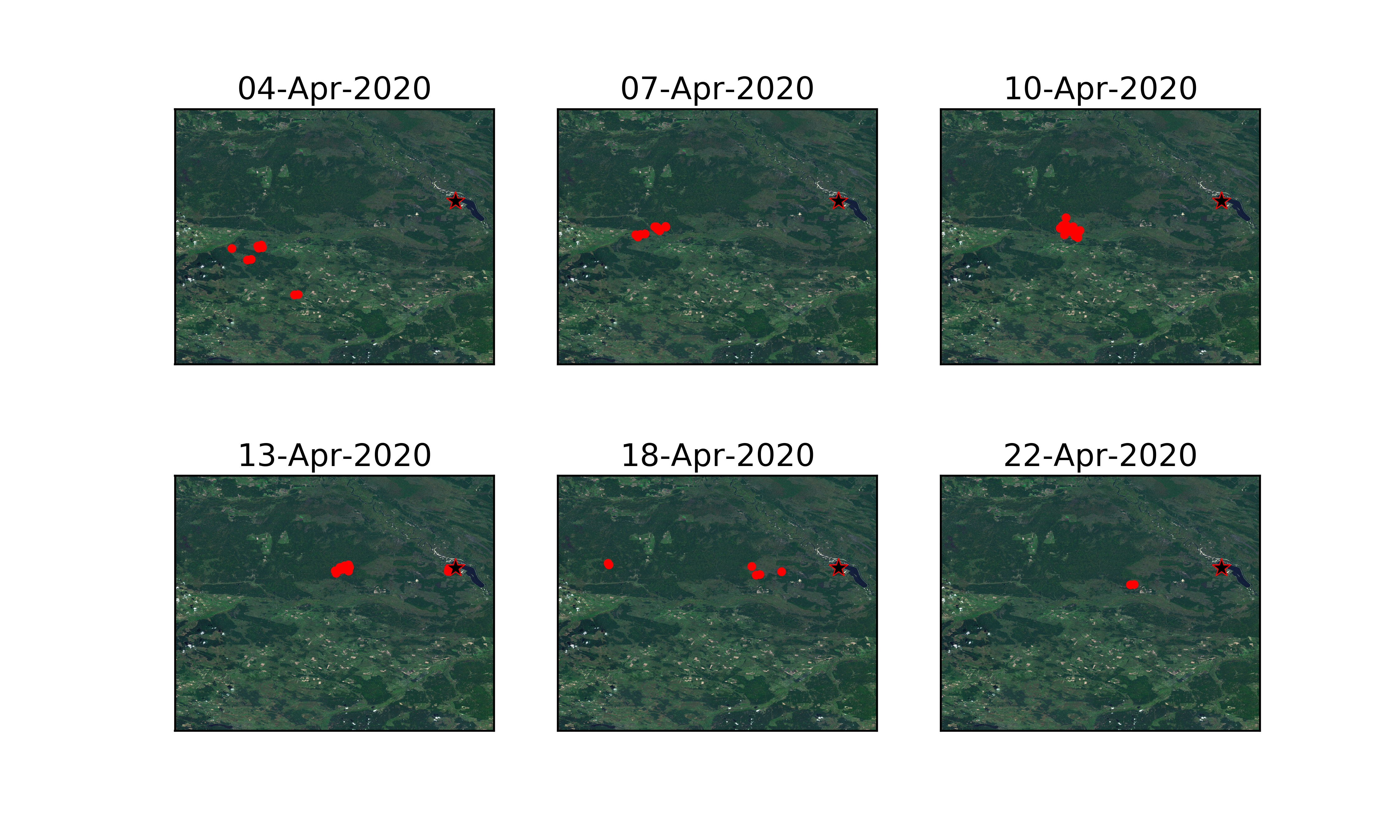


Fig. S 2: MOD14/MYD14 thermal anomalies from MODIS based on Terra and Aqua satellites retrieved for the period 1 – 22 April 2020 in Chernobyl. Only active fires with maximum confidence (100%) were used, in order to assure that only real fires are taken into account. A selection of six days is only shown. The largest number of detection was observed on April 9^th^ and 10^th^ ^2^. The star denotes the location of the Chernobyl Nuclear Power Plant (NPP). Maps have been generated with the open access module matplotlib^23^ (license: <https://matplotlib.org/3.2.1/users/license.html>).

The contamination levels of ^137^Cs, ^90^Sr, ^238^Pu, ^239^Pu, ^240^Pu and ^241^Am in the specific regions suffering from the April 2020 fires were retrieved from Evangeliou et al.^5^ following detailed measurements^6–8^ (available in <https://radio.nilu.no>). Maps of gridded contamination levels can be found in **Fig. S 3**. To our knowledge, there is no detailed study on radioactivity measurement in biomass from the Chernobyl Exclusion Zone (CEZ). As regards to radionuclide emission factors (EFs), old studies report that about 20% of labile radionuclides can be remobilised after fires from soil ^9^ and up to 40–100% from biomass/vegetation^10,11^, while more recent ones show that these EFs are lower (4–10%)^12,13^. Recently, Hao et al.^14^ updated these EFs for ^137^Cs in laboratory experiments and found that only 1–2.5% of ^137^Cs in the biomass could be redistributed. As for ^90^Sr and the refractory radionuclides, very few measurement of emission factors from biomass burning exist, all suggesting very small amount to be rersuspended^13,15^. Here, we used an EF of 1.2% for ^137^Cs, 0.2% for ^90^Sr and 0.1% for ^238^Pu, ^239^Pu, ^240^Pu and ^241^Am. The radionuclide emissions from this method (**Fig. S 4**) were calculated as the spatial deposition density of the radionuclides (Bq m^-2^) multiplied with the MODIS-based burned area (m^2^) and applying the aforementioned EFs.


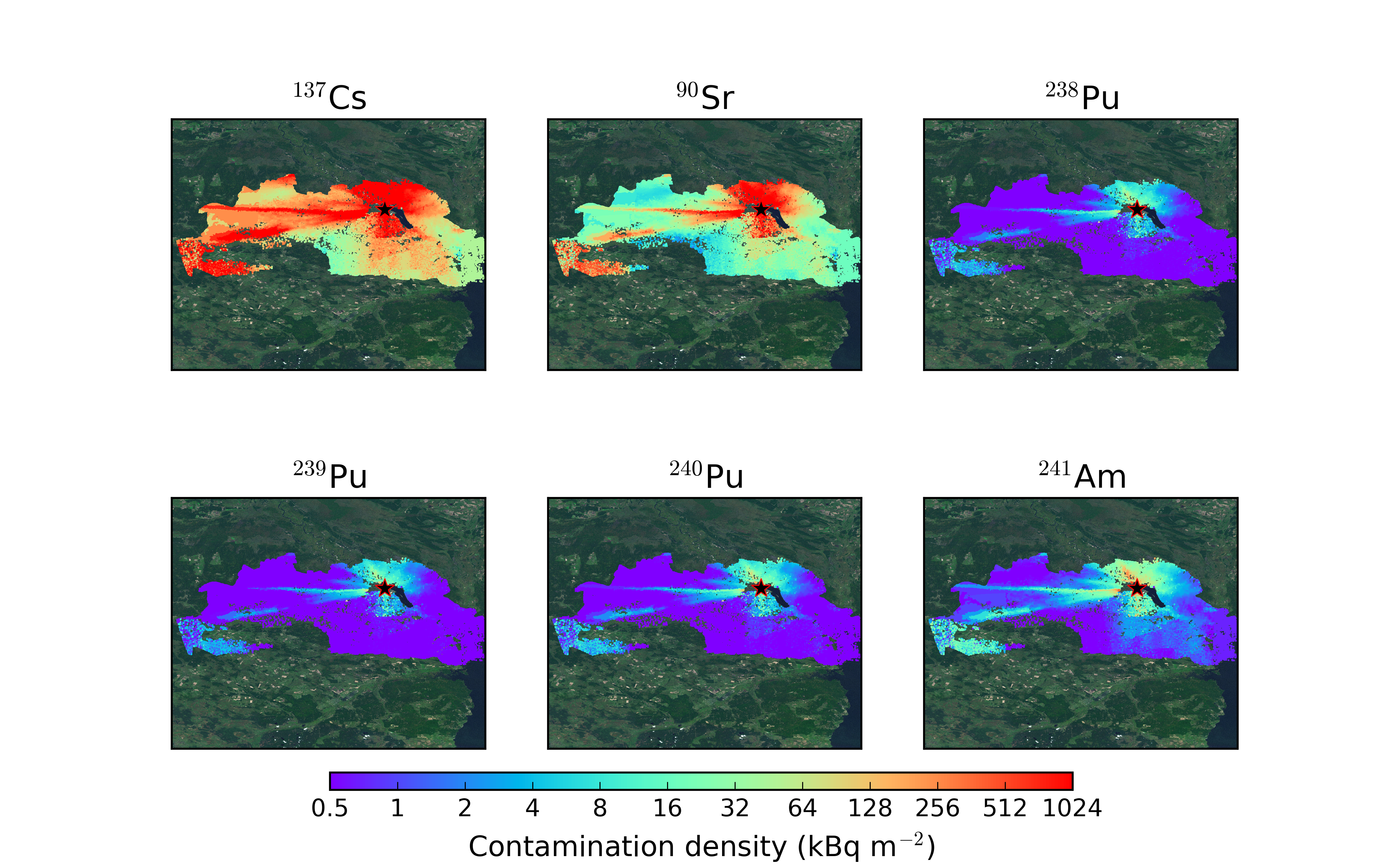


Fig. S 3: Spatial contamination density of ^137^Cs, ^90^Sr, ^238^Pu, ^239^Pu, ^240^Pu and ^241^Am in the CEZ corrected for physical and ecological decay to April 2020 ^16^. All measurements are publicly available from <https://radio.nilu.no> and are based on numerous measurements that have taken place after the Chernobyl accident in April 1986 ^5–8^. The star denotes the location of the Chernobyl Nuclear Power Plant (NPP). Maps have been generated with the open access module matplotlib^23^ (license: <https://matplotlib.org/3.2.1/users/license.html>).


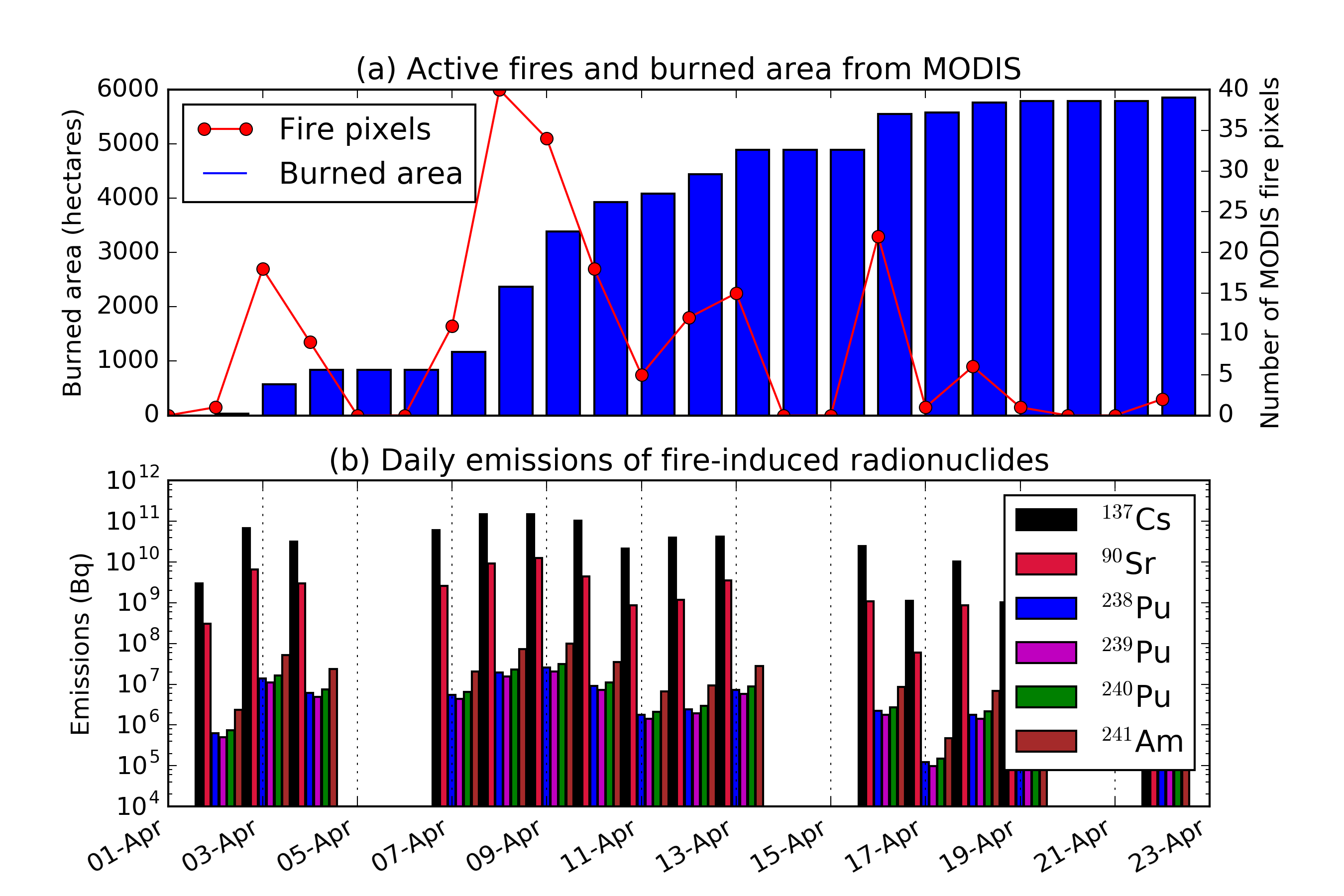


Fig. S 4: (a) Active fire counts per day in the CEZ from MODIS MOD14/MYD14 products and accumulated burned area calculated with the modified method of Stohl et al.^3^ According to this, around 60 thousand hectares (600 km^2^) must have been burned in the CEZ. (b) Daily emissions of ^137^Cs, ^90^Sr, ^238^Pu, ^239^Pu, ^240^Pu and ^241^Am from the April 2020 fires in the CEZ.

**Method 2: Emissions derived from CAMS GFAS combusted biomass combined with radionuclide EFs.** CAMS GFAS assimilates fire radiative power (FRP) observations from satellite-based sensors converting the energy released during fire combustion into gases and aerosol daily fluxes^17,18^. Data are available globally on a regular grid with horizontal resolution of 0.1 degrees from 2003 to present. FRP observations assimilated in GFAS are the NASA Terra MODIS and Aqua MODIS active fire products (<http://modis-fire.umd.edu/>)^19^. FRP measures the heat power emitted by fires, as a result of the combustion process and is directly related to the total biomass combusted^20^. Using land-use dependent conversion factors, GFAS converts FRP into emission estimates of 44 smoke constituents, such as CO, CO_2_, CH_4_, black-carbon and organic matter components of the aerosols^18^. We use the total combusted biomass (**Fig. S 5**a) and then apply an EF for ^137^Cs from Hao et al.^14^ for duff and pine-needle vegetation. Similar to the previous method, we used identically low EFs for ^90^Sr and ^238^Pu, ^239^Pu, ^240^Pu and ^241^Am following the previous method.


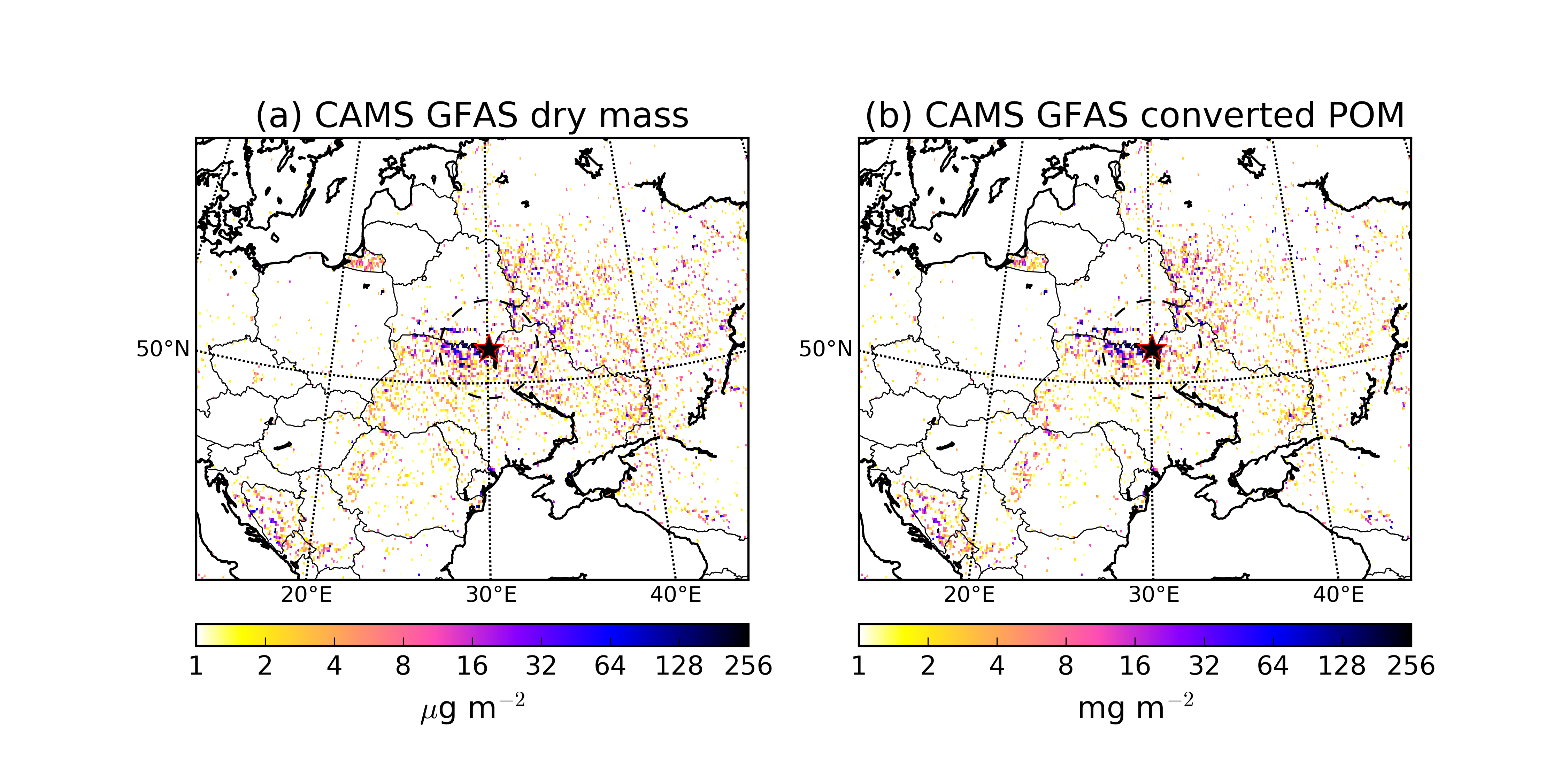


Fig. S 5: (a) Dry combusted mass summed from 1 – 22 April 2020. FRP was retrieved from CAMS GFAS and was converted to dry combusted mass with the method of Wooster et al.^20^ (b) POC retrieved from CAMS GFAS in spatial resolution of 0.1°×0.1° and summed for the same period (1 – 22 April 2020). POC was converted to POM as in Strode et al.^21^ The star shows the location of the Chernobyl Nuclear Power Plant (CNPP). It is evident that both dry combusted mass and POM emissions are maximum in the vicinity of the CNPP, due to large vegetation cover that has been observed there ^22^. The dashed line represents the CEZ. Maps have been generated with the open access module matplotlib^23^ (license: <https://matplotlib.org/3.2.1/users/license.html>).

**Method 3: Emissions derived from CAMS GFAS particulate organic carbon combined with radionuclide EFs.** One of the 44 smoke constituents that are delivered by CAMS GFAS is particulate organic carbon (POC). Strode et al.^21^ found that MODIS aerosol optical depth coincide with detections of ^137^Cs and used the relationship between ^137^Cs and aerosols to model ^137^Cs released from the summer 2010 wildfires in Russia based on organic carbon emissions. In their model, they used a constant factor of 1.4 to convert organic carbon to particulate organic matter (POM) (**Fig. S 5**b). Based on measurements from the International Monitoring System (IMS) for the detection of airborne radioactivity designed and implemented for the Comprehensive Nuclear-Test-Ban Treaty Organisation (CTBTO), they calculated a ^137^Cs/POM ratio equal to 0.23 kBq kg^-1^, assuming that particles containing ^137^Cs undergo the same behavior in physicochemical processes.

With this method, they managed to reproduce the timing of many of the observed peaks at measurement sites capturing much of the impact of biomass burning on ^137^Cs concentrations. Of course, this method has numerous limitations, such bias on the satellite aerosol optical depth used to define strength or location of biomass burning aerosol emissions. Furthermore, the ratio of ^137^Cs/POM is expected to vary regionally depending on the contamination levels of ^137^Cs in soil and vegetation. Knowing the exact contamination levels of ^137^Cs over Europe from the Chernobyl era, hence in Western Russia, and using publicly available data (<https://radio.nilu.no>) corrected for physical and ecological decay^16^, we adapted this ratio in the CEZ assuming that Western Russia and Northern Ukraine are more or less the same boreal environments (hence, the same amount of POM will be released from vegetation fires). Like in the previous methods, we used identical radionuclide/POM ratios for ^90^Sr and the refractory radionuclides.

**Relevant statistical tests used:** To perform a detailed and statistically significant comparison between model predicted concentrations and observations, we calculated the mean fractional bias (MFB) as follows:

$$MFB=\frac{1}{N}\sum_{i=1}^{N} (\frac{C_{m}-C_{o}}{\frac{C_{m}+C_{o}}{2}})\times100\%$$

where $C_{m}$ and $C_{o}$ are the modelled and measured concentrations of ammonia and $N$ is the total number of observations (24). MFB is a symmetric performance indicator that gives equal weights to under- or over-estimated concentrations (minimum to maximum values range from -200% to 200%). Furthermore, we assess how concentrated the data is around the line of best fit using the root mean square error (RMSE) as follows:

$$RMSE=\sqrt{\sum_{i=1}^{N} \frac{{{(C}_{m}-C_{o})}^{2}}{N}}$$

**Calculation of internal and external effective doses.** Dose-rates were calculated for exposure pathways of deposition and air-submersion (external pathways) and inhalation (internal pathway). Food and water ingestion were omitted due to the lack of information. The methodology was adopted from WHO^1^ report on Fukushima for 1-year old infants, 10-year old children, and adult and from ICRP^24^ for firefighters.

The effective dose-rate from deposition is given by the following equation:

$$E_{i}^{dep}\left( t \right)=r\left( t \right)[\sum_{m} A_{m}.d_{m}^{dep} e^{-\lambda_{m}t}] {RF}_{i}$$

where the summation index m is for each of the deposited radionuclides, $\lambda_{m}$ is the decay constant of radionuclide m (0.023 y^-1^ for ^137^Cs), $d_{m}^{dep}$ is the dose rate coefficient from surface activity density to kerma rate in free air (^137^Cs+^137m^Ba: 1.69, 1.34 and 1.25 nSv h^-1^ per kBq m^-2^ for 1-year old infant, 10-year old child, and adults/firefighters), $A_{m}$ is the surface activity density of radionuclide m on the ground (Bq m^-2^), ${RF}_{i}$ is a reduction factor based on occupancy of the population (assumed to be 0.6 for adults) and $r\left( t \right)$ is a time dependent attenuation factor that accounts for radionuclide penetration in the soil (for the first 6 months after releases the minimum value is 0.925). Radionuclides emitting alpha and beta particles had one order of magnitude lower dose conversion factors and, threrefore, they were not considered.

The effective dose-rate from air-submersion is calculated as follows:

$$E_{i}^{air}=[\sum_{m} A_{m}.d_{m}^{air}]\tau_{exp} {RF}_{i}$$

where $d_{m}^{air}$ is the dose rate coefficient from a semi-infinite volume source in air to kerma in free air for a height of 1 m above ground due to uniform distribution ($A_{m}$) of radionuclide m in the air (^137^Cs+^137m^Ba: 0.118, 0.097 and 0.092 nSv h^-1^ per Bq m^-3^ for 1-year old infant, 10-year old child, and adults/firefighters) and $\tau_{exp}$ is the exposure time (we assume it is 24 h accounting for a reduction due to occupancy). Radionuclides emitting alpha and beta particles had one order of magnitude lower dose conversion factors and, threrefore, they were not considered. The effective dose from air–submersion is integrated over the period where concentrations are above zero.

Finally, the effective dose from inhalation of radioactive materials was calculated according to:

$$E_{i}^{inh}=I [\sum_{m} \frac{A_{m}}{V_{bm}}d_{m}^{inh}]$$

where $I$ is the breathing rate for population group (2.57×10^-4^ m^3^ s^-1^ for adult/workers/firefighters, 1.77×10^-4^ m^3^ s^-1^ for10-year old children and 0.602×10^-4^ m^3^ s^-1^ for 1-year old infants), $d_{m}^{inh}$ is the effective dose inhalation coefficient for adults and for radionuclide m (^137^Cs+^137m^Ba: 4.6, 3.7, 5.4, 4.8 (6.7)×10^-9^ Sv Bq-1, ^90^Sr: 1.5×10^-7^, 1.1×10^-7^, 3.6×10^-8^ (2.4–3.0×10^-8^), ^238-240^Pu: 7.8×10^-5^, 7.4×10^-5^, 4.6×10^-5^ (3.0–4.3×10^-5^), ^241^Am: 7.3×10^-5^, 6.9×10^-5^, 4.2×10^-5^ (2.7–3.9×10^-5^) for 1-year old infant, 10-year old child, adult and firefighters for 1(5) μm particles) and $V_{bm}$ is bulk deposition velocity of radionuclide m for particular weather and surface conditions ( m s^-1^).

Table S 1: Sparse observations of surface activity concentrations of ^137^Cs reported in April 2020 ^25–28^ after the wildfires in the CEZ. The results are based on filter sampling followed by gamma spectrometric measurements.

|  | Longitude | Latitude | Date | ^137^Cs  (μBq m^-3^) |
| --- | --- | --- | --- | --- |
| Central Geophysical Observatory (Kiev) | 30.532105 | 50.392199 | 6 Apr-20 | 83 |
| ---------- « ---------- | 30.532105 | 50.392199 | 7 Apr-20 | 110 |
| ---------- « ---------- | 30.532105 | 50.392199 | 8 Apr-20 | 140 |
| ---------- « ---------- | 30.532105 | 50.392199 | 9 Apr-20 | 290 |
| ---------- « ---------- | 30.532105 | 50.392199 | 10 Apr-20 | 140 |
| ---------- « ---------- | 30.532105 | 50.392199 | 11 Apr-20 | 700 |
| ---------- « ---------- | 30.532105 | 50.392199 | 12 Apr-20 | 170 |
| ---------- « ---------- | 30.532105 | 50.392199 | 13 Apr-20 | 180 |
| Ukrainian Hydrometeorological Institute (Kiev) | 30.53341 | 50.392004 | 7 Apr-20 | 57 |
| ---------- « ---------- | 30.53341 | 50.392004 | 8 Apr-20 | 65 |
| ---------- « ---------- | 30.53341 | 50.392004 | 9 Apr-20 | 220 |
| ---------- « ---------- | 30.53341 | 50.392004 | 10 Apr-20 | 470 |
| ASKRS Chernobyl | 30.21772 | 51.2889 | 10 Apr-20 | 240 |
| ASKRS Kids | 30.2397 | 51.1986 | 8 Apr-20 | 54 |
| ASKRS Kopachi | 30.08 | 51.36 | 11 Apr-20 | 1000 |
| ASKRS Kopachi | 30.08 | 51.36 | 8 Apr-20 | 72 |
| ASKRS GRP-750 | 30.3159 | 51.3078 | 8 Apr-20 | 630 |
| Chernobyl, vul. Kirov, 42 | 30.217 | 51.275 | 11 Apr-20 | 2600 |
| Chernobyl, Vul. school, 6 | 30.217 | 51.275 | 12 Apr-20 | 290 |
| Korohods'kyi Peninsula 12 kv | 30.162 | 51.3579 | 12 Apr-20 | 42000 |
| Korogodske L-in 41 m2 | 30.0326 | 51.2937 | 12 Apr-20 | 1600 |
| Chernobyl NPP Ukrenergomontazh  GRP- 750 | 30.217 | 51.275 | 13 Apr-20 | 180000 |
| Vienna International Centre (VIC) | 16.4165 | 48.2355 | 7-8 Apr-20 | 3.75 |
| Thessaloniki (Greece) | 22.9444 | 40.6401 | 11-13 Apr-20 | 20 |
| Orsay (France) | 2.185 | 48.704 | 1-8 Apr-20 | 0.139 |
| Orsay (France) | 2.185 | 48.704 | 8-15Apr-20 | 0.267 |
| Dijon (France) | 5.041 | 47.32 | 30 Mar-20 – 6 Apr-20 | 0.0184 |
| Romagnat (France) | 3.086 | 47.718 | 3-9 Apr-20 | 0.269 |
| Revin (France) | 4.69 | 49.94 | 1-16 Apr-20 | 0.183 |
| Bouc Bel Air (France) | 5.406 | 43.448 | 4-10 Apr-20 | 1.161 |
| Bugey (France) | 5.267 | 45.802 | 30 Mar-20 – 6 Apr-20 | 2.7 |
| Nancy (France) | 6.177 | 48.688 | 31 Mar-20 – 7 Apr-20 | 2.5 |
| Fessenheim (France) | 7.528 | 47.909 | 30 Mar-20 – 6 Apr-20 | 0.7 |
| Grenoble (France) | 5.724 | 45.178 | 30 Mar-20 – 6 Apr-20 | 0.44 |

Table S 2: Different mass fractions used per particle size for the sensitivity analysis of the transport of ^137^Cs from the April 2020 fires in the CEZ. Mass fraction is the percentage of total mass associated with each of the mean particle size used in the model. Emphasis on larger particles (>10 μm) is given following Hao et al.^14^ findings.

|  | <2.5 μm | 2.5–10 μm | >10 μm |
| --- | --- | --- | --- |
| Mass fraction 1 | 20% | 20% | 60% |
| Mass fraction 2 | 10% | 20% | 70% |
| Mass fraction 3 | 10% | 10% | 80% |
| Mass fraction 4 | 0% | 10% | 90% |
| Mass fraction 5 | 0% | 0% | 100% |
| Mass fraction 6 | 0% | 40% | 60% |
| Mass fraction 7 | 0% | 30% | 70% |
| Mass fraction 8 | 0% | 20% | 80% |
| Mass fraction 9 | 10% | 0% | 90% |
| Mass fraction 10 | 20% | 0% | 80% |

Table S 3. Different emissions factors (EFs)^10–14^ used is the sensitivity analysis for the transport of ^137^Cs from the April 2020 fires in the CEZ. These EFs are based on laboratory and open field experiments for different types of vegetation and burning temperatures.

|  | Percentage fraction emitted | Reference |
| --- | --- | --- |
| EF 1 | 60% (20–100%) | Amiro et al.^10^ |
| EF 2 | 35% (10–40%) | Horrill et al.^11^ |
| EF 3 | 10% | Piga^12^ |
| EF 4 | 4% | Yoschenko et al.^13^ |
| EF 5 | 1.2% (1–2.5%) | Hao et al.^14^ |

Video 1: Surface atmospheric activity concentrations (3-hourly) of ^137^Cs, ^90^Sr, ^238^Pu, ^239^Pu, ^240^Pu and ^241^Am released from the April 2020 wildfires in the CEZ over Europe. Units of μBq m^-3^ were used in the plot, because the typical limit of detection for airborne ^137^Cs is a few tens of μBq m^-3^ depending on sampling and measuring time^23,24^. The dashed cycle shows the CEZ. Maps have been generated with the open access module matplotlib^64^ (license: <https://matplotlib.org/3.2.1/users/license.html>).

Video 2: 3-hourly cumulative deposition of ^137^Cs, ^90^Sr, ^238^Pu, ^239^Pu, ^240^Pu and ^241^Am over Europe as a result of resuspension and transport during April 2020 fires in the CEZ (shown with a dashed cycle). The maps show deposition in logarithmic scale from μBq m^-2^ to mBq m^-2^. However, the typical limit of detection for ground deposition is about one order of magnitude higher^65^ making deposition practically unmeasurable outside the CEZ. Maps have been generated with the open access module matplotlib^64^ (license: <https://matplotlib.org/3.2.1/users/license.html>).

**References**

1. WHO. Preliminary dose estimation from the nuclear accident after the 2011 Great East Japan Earthquake and Tsunami. *WHO* (2012). Available at: https://apps.who.int/iris/bitstream/handle/10665/44877/9789241503662_eng.pdf;jsessionid=C841958E3309A981786A745C052B34C9?sequence=1. (Accessed: 30th April 2020)

2. Giglio, L., Descloitres, J., Justice, C. O. & Kaufman, Y. J. An enhanced contextual fire detection algorithm for MODIS. *Remote Sens. Environ.* **87**, 273–282 (2003).

3. Stohl, A. *et al.* Arctic smoke &ndash; record high air pollution levels in the European Arctic due to agricultural fires in Eastern Europe in spring 2006. *Atmos. Chem. Phys.* **7**, 511–534 (2007).

4. Wotawa, G. *et al.* Inter- and intra-continental transport of radioactive cesium released by boreal forest fires. *Geophys. Res. Lett.* **33**, 4–7 (2006).

5. Evangeliou, N. *et al.* Reconstructing the Chernobyl Nuclear Power Plant (CNPP) accident 30 years after. A unique database of air concentration and deposition measurements over Europe. *Environ. Pollut.* (2016). doi:10.1016/j.envpol.2016.05.030

6. Kashparov, V. A. *et al.* Territory contamination with the radionuclides representing the fuel component of Chernobyl fallout. *Sci. Total Environ.* **317**, 105–119 (2003).

7. Kashparov, V. *et al.* Spatial datasets of radionuclide contamination in the Ukrainian Chernobyl Exclusion Zone. 339–353 (2018).

8. Kashparov, V. A. *et al.* Soil contamination with 90Sr in the near zone of the Chernobyl accident. *J. Environ. Radioact.* **56**, 285–298 (2001).

9. Paliouris, G., Taylor, H. W., Wein, R. W., Svoboda, J. & Mierzynski, B. Fire As an Agent in Redistributing Fallout Cs-137 in the Canadian Boreal Forest. *Sci. Total Environ.* **160**–**61**, 153–166 (1995).

10. Amiro, B. D., Sheppard, S. C., Johnston, F. L., Evenden, W. G. & Harris, D. R. Burning radionuclide question: What happens to iodine, cesium and chlorine in biomass fires? *Sci. Total Environ.* **187**, 93–103 (1996).

11. Horrill, A. D., Kennedy, V. H., Paterson, I. S. & McGowan, G. M. The effect of heather burning on the transfer of radiocaesium to smoke and the solubility of radiocaesium associated with different types of heather ash. *J. Environ. Radioact.* **29**, 1–10 (1995).

12. Piga, D. Processus engagés dans la rémanence, au niveau du compartiment atmosphérique, des radionucléides artificiels antérieurement déposés. (2010).

13. Yoschenko, V. I. *et al.* Resuspension and redistribution of radionuclides during grassland and forest fires in the Chernobyl exclusion zone: Part I. Fire experiments. *J. Environ. Radioact.* **86**, 143–163 (2006).

14. Hao, W. M. *et al.* Cesium emissions from laboratory fires. *J. Air Waste Manage. Assoc.* **68**, 1211–1223 (2018).

15. Yoschenko, V. I. *et al.* Resuspension and redistribution of radionuclides during grassland and forest fires in the Chernobyl exclusion zone: Part II. Modeling the transport process. *J. Environ. Radioact.* **87**, 260–278 (2006).

16. Evangeliou, N. *et al.* Resuspension and atmospheric transport of radionuclides due to wildfires near the Chernobyl Nuclear Power Plant in 2015: An impact assessment. *Sci. Rep.* **6**, 26062 (2016).

17. Giuseppe, F. Di, Remy, S., Pappenberger, F. & Wetterhall, F. Improving GFAS and CAMS biomass burning estimations by means of the Global ECMWF Fire Forecast system (GEFF). *ECMWF Tech. Memo.* (2016).

18. Kaiser, J. W. *et al.* Biomass burning emissions estimated with a global fire assimilation system based on observed fire radiative power. *Biogeosciences* **9**, 527–554 (2012).

19. Kaufman, Y. J. *et al.* Fire and smoke observed from the earth observing system MODIS instrument - Products, validation, and operational use. *Int. J. Remote Sens.* **24**, 1765–1781 (2003).

20. Wooster, M. J., Roberts, G., Perry, G. L. W. & Kaufman, Y. J. Retrieval of biomass combustion rates and totals from fire radiative power observations: FRP derivation and calibration relationships between biomass consumption and fire radiative energy release. *J. Geophys. Res. Atmos.* **110**, 1–24 (2005).

21. Strode, S. A., Ott, L. E., Pawson, S. & Bowyer, T. W. Emission and transport of cesium-137 from boreal biomass burning in the summer of 2010. *J. Geophys. Res. Atmos.* **117**, 1–8 (2012).

22. Ager, A. A. *et al.* The wildfire problem in areas contaminated by the Chernobyl disaster. *Sci. Total Environ.* **696**, 133954 (2019).

23. Hunter, J. D. Matplotlib: A 2D Graphics Environment. *Comput. Sci. Eng.* **9**, 90–95 (2007).

24. ICRP. *ICRP Publication 119: Compendium of Dose Coefficients based on ICRP Publication 60*. *Annals of the ICRP* **41**, (Elsevier Ltd, 2012).

25. IRSN. Information note Fires in Ukraine in the exclusion zone around the Chernobyl power plant : Point position. 1–9 (2020). Available at: https://www.irsn.fr/EN/newsroom/News/Documents/IRSN_Information-Report_Fires-in-Ukraine-in-the-Exclusion-Zone-around-chernobyl-NPP_15042020.pdf. (Accessed: 29th April 2020)

26. Greek Atomic Energy Commission. Measurement results in Greece related to the forest fire in the area of Chernobyl, Ukraine. (2020). Available at: http://eeae.gr/en/news/announcements/measurement-results-in-greece-related-to-the-forest-fire-in-the-area-of-chernobyl,-ukraine. (Accessed: 29th April 2020)

27. Zerbo, L. Twitter. (2020). Available at: https://twitter.com/SinaZerbo/status/1250149680450854915/photo/1. (Accessed: 29th April 2020)

28. IRSN. Fires in Ukraine in the exclusion zone around the Chernobyl power plant : First results of 137 Cs measurements in France. 1–4 (2020). Available at: https://www.irsn.fr/EN/newsroom/News/Documents/IRSN_Information-Report_Fires-in-Ukraine-in-the-Exclusion-Zone-around-chernobyl-NPP_24042020.pdf. (Accessed: 2nd May 2020)
